# Performance of p16/Ki67 immunostaining, HPV E6/E7 mRNA testing, and HPV DNA assay to detect high-grade cervical dysplasia in women with ASCUS

**DOI:** 10.1186/s12885-019-5492-9

**Published:** 2019-03-27

**Authors:** Yuanhang Zhu, Chenchen Ren, Li Yang, Xiaoan Zhang, Ling Liu, Zhaoxin Wang

**Affiliations:** 1 Department of Obstetrics and Gynecology, The Third Affiliated Hospital of Zhengzhou University, No. 7, Front Kangfu Street, Zhengzhou, 450052 Henan Province People’s Republic of China; 2 Department of Imaging, The Third Affiliated Hospital of Zhengzhou University, Zhengzhou, 450052 People’s Republic of China

**Keywords:** HPV, E6/E7, mRNA, p16/Ki67, ASCUS, CIN

## Abstract

**Background:**

Atypical squamous cell of undetermined significance (ASCUS) is a common cervical cytological diagnosis. At present, HPV DNA assay is used to triage these patients, but its lower specificity brings a series of problems. The purpose of this study was to evaluated the value of p16/Ki67 immunostaining, HPV E6/E7 mRNA testing in triaging women with ASCUS by comparing HPV DNA assay.

**Methods:**

Liquid based cytology specimens were collected from 300 patients. P16/Ki67 immunocytochemistry using the CINtec® Plus Kit and HPV E6/E7 mRNA testing by QuantiVirus®HPV E6/E7 mRNA assay used the same cytology sample. Detection rates of each test were evaluated against histopathology.

**Results:**

All assays yielded a high sensitivity for the detection of CIN3+ (100% (86.7–100) for HPV DNA assay, 88.0% (70.0–95.8) for HPV E6/E7 mRNA testing and 100% (86.7–100) for p16/Ki67 immunocytochemistry) and CIN2+ (98.2% (90.2–99.7) for HPV DNA assay, 87.0% (75.6–93.6) for HPV E6/E7 mRNA testing, 98.2% (90.2–99.7) for p16/Ki67 immunocytochemistry). The specificity to detect high grade dysplasia was highest for p16/Ki67 immunocytochemistry (74.2% (68.7–79.0) in CIN3+ and 82.5% (77.3–86.8) in CIN2+), followed by HPV E6/E7 mRNA testing (39.6% (34.0–45.5) in CIN3+ and 42.7% (36.7–48.9) in CIN2+) and HPV DNA assay (16.0% (12.1–20.8) in CIN3+ and 17.5% (13.2–22.7) in CIN2+).

**Conclusions:**

p16/Ki67 immunostaining and HPV E6/E7 mRNA testing, especially the former, may be promising tools in triage of ASCUS.

## Background

Cervical cancer screening based on the Papanicolaou (Pap) smear is widely accepted as the most important public health strategy and have reduced cervical cancer incidence and mortality during the last decades in many countries [1]. The goal in primary screening for cervical cancer is to detect and treat high-grade cervical intraepithelial lesions before invasive cancer develops [2]. However, the Pap test has a low single-test sensitivity for detection of high grade cervical disease and requires extensive training and experience, which may generate many equivocal results, atypical squamous cell of undetermined significance (ASCUS), that require repeated testing and further workup and thus generates high costs in population-based screening [3]. Unfortunately, Statistical analysis shows that ASCUS is the most frequent abnormal cervical cytology, with 59.3% of abnormal Pap smear result [4], but the risk of CIN2+ is only 9.7% [5]. Virtually all cervical cancers are caused by persistent infections with high-risk human papillomaviruses (HR-HPV), which led the development of HPV DNA test to improve the cervical cancer screening strategies [6]. Based on current recommendations, HPV DNA testing is used as an adjunctive test for all cervical cytology samples (co-testing) in women over 30 years old [7, 8], and has been approved as a triage test for women with ASCUS [3]. With further in-depth research, we found that it was generally not considered effective to triage ASCUS by using HPV DNA assay because of lack of specificity for high-grade cervical intraepithelial lesions [5].

Recently, many disease-specific molecular markers of cervical cancer have been recognized based on our recognition of HPV-related carcinogenesis. Only the persistent infection and malignant transformation in cervix can cause the occurrence and development of cervical malignant transformation [9]. The most important factor in cervical carcinogenesis progression is certainly the integration of HPV sequences into the host genome with the loss of E2 tumor suppressor gene. E2 physiologically regulates the expression of E6 and E7 oncogenes. p16^INK4a^ (p16) is a tumor suppressor protein playing a crucial role in cell-cycle regulation. p16-overexpression is considered as a surrogate marker for deregulated E7 expression and hence for transforming HPV infections [10, 11]. Ki67 is a well-known cell proliferation marker, useful for confirmation of the diagnosis in ambiguous cases [12, 13]. Under physiological conditions, the co-expression of p16 and Ki67 protein does not occur, since they typically induce opposite effects [14]. Many disease-specific molecular markers may base on the direct or indirect detection of the viral oncogene E6 and E7 expression in HPV transformed basal keratinocytes. The accuracy of cervical cancer screening programs may be improved by biomarker assays that specifically highlight transforming HPV infections [15].

HPV E6/E7 mRNA test has been proposed as a biomarker for HPV oncogene expression. Previous studies showed HPV E6/E7 mRNA test has a very good clinical sensitivity and higher specificity compared to HPV DNA detection [16–18]. p16/Ki67 immunocytochemistry has been proved to be a surrogate marker for the prediction of high-risk precursor or invasive cervical cancer lesions [15]. The accuracy of p16/Ki67 immunocytochemistry and HPV E6/E7 mRNA test for triaging ASCUS has been analyzed in few studies so far [19, 20], however, who is the better one to triage ASCUS has not been determined. Here, we evaluated the diagnostic accuracy of p16/Ki67 immunocytochemistry, HPV E6/E7 mRNA testing and HPV DNA assay by a cross-sectional study.

## Methods

### Study population and study design

The study population consisted of women who attended outpatient gynecological screening at the gynecology department of The Third Affiliated Hospital of Zhengzhou University between 2015 and 2017. Women meeting these conditions are enrolled in our study:(1) Cytological diagnosis for ASCUS; (2) non-pregnancy; (3) No immunodeficiency disease; (4) No cervical surgery or chemo and/or radiotherapy for cervical malignant disease. A total of 300 women were consecutively included and met those conditions in this study. All women underwent Pap test, colposcopy biopsy and histopathological examination. After the Pap test was done, the residual LBC specimen was used for assays evaluated in this study, HPV E6/E7 mRNA and p16/Ki67 immunocytochemistry. The Ethics Committee of The Third Affiliated Hospital of Zhengzhou University reviewed and approved our study. All enrolled women were aware of the research purposes and signed the consent forms. The median age was 36 years (interquartile range 18–70 years).

### Liquid based cytology

LBC technology was used to perform Cytological detection. We used Thin Prep 2000 Processor (Cytyc Corporation, Marlborough, MA, USA) to make Thinlayer slides according to the manufacturer’s instructions. Two experienced cytopathologists independently diagnosed cytological specimens at the same time without knowing the results of the other assays. If the two cytopathologists gave different diagnosis, cervical samples were reviewed again. Using the 2001 Bethesda Reporting System Criteria to report the diagnosis of cytological specimens [21]. The analysis of p16/Ki67 immunocytochemistry in cytological categories is based on the same Pap sample.

### p16/Ki67 immunocytochemistry

Cytology slide for p16/Ki67 immunocytochemistry was prepared from the residual LBC specimen using a T2000 slide processor (Hologic, Bedford, MA, USA). We purchased CINtec® Plus Kit (Roche mtm laboratories AG, Heidelberg, Germany) to carry immunocytochemistry of p16/Ki67 according to the manufacturer’s instructions. An experienced cytotechnologist reviewed all cervical cytology slides for detecting the staining performance of two markers. If ≥1 cervical epithelial cell stained both with a brown cytoplasmic stain (p16) and a red nuclear (Ki67) no matter how abnormal the morphology of the cells were, this case was considered as positive of p16/Ki67 immunocytochemistry. Slides without brown cytoplasmic stain and /or a red nuclear stain were called negative [22].

### HPV E6/E7 mRNA testing

Residual Pap specimens were also used to detect the 14 types of HR-HPV E6/E7 mRNA by QuantiVirus®HPV E6/E7 mRNA assay (Kodia, Henan, China) according to the manufacturer’s instructions. The assay used branched DNA (b-DNA) technology (DiaCarta, CA, USA), which don’t need the step of RNA purification or RT-PCR to quantitatively detect HPV mRNA. In each sample, the result of mRNA was marked as light unit. Special calculation software can convert light unit to copy number under the fact of light emission related directly to the amount of HPV mRNA. If the copy number was equal or greater than 1.0, the result of E6/E7 mRNA was positive. If less than 1.0, negative is the result [23].

### HPV DNA assay

Hybrid Capture 2 assay (HC2, Digene, Gaithersburg, MD, USA) was used to detect 13 types of HR-HPV DNA according to the manufacturer’s instructions. If the number of RLU/CO equal or greater than 1.0, the result of HPV DNA was positive.

### Colposcopy and histological diagnosis

Women with ASCUS underwent colposcopy biopsy within 4 weeks after cervical exfoliative cytology test. In this study, some patients with ASCUS but a negative HR-HPV DNA result required to do colposcopy biopsy because of fear of suffering from cervical malignant disease, thus we underwent colposcopy biopsy for them. Two experienced pathologists independently diagnosed histological slides without knowing the results of the other assays. If the two pathologists gave different diagnosis, histological slides were diagnosed again for a consensus result. The diagnosis result of histological slides meets the standard of current World Health Organization classification. The diagnoses of cervical inflammation and mild cervical intraepithelial neoplasia (Grade 1 cervical intraepithelial neoplasia, CIN1) are referred as CIN1-. cervical inflammation, CIN1 and Moderate cervical intraepithelial neoplasia (Grade 2 cervical intraepithelial neoplasia, CIN2) are referred as CIN2-. CIN2, severe cervical intraepithelial neoplasia (Grade 3 cervical intraepithelial neoplasia, CIN3) and carcinoma are referred as CIN2 +. CIN3 and carcinoma are referred as CIN3+. In this study, all histological specimens were subjected to p16-immunohistochemistry. The staining was performed on 2 μm sections from formalin fixed, paraffin embedded tissues according to the manufacturer’s instructions using the CINtec p16^INK4a^-histology kit (mtm Laboratories, Heidelberg, Germany). For CIN2, it diagnosed clinically as CIN2 when p16-histology positive, diagnosed clinically as CIN1 when p16-histology negative.

### Statistics

SPSS 21.0 software was used to perform statistical analysis. Wilcoxon rank sum test of two independent samples was used to compare the differences of the expression levels of HPV E6/E7 mRNA between different groups. Chi-square tests were used to compare the differences of percentage between different groups. The gold standard for cervical disease was histologically confirmed CIN2+ and CIN3+. Diagnostic sensitivity and specificity for the detection of CIN2+ and CIN3+ was calculated for all assays based on 2 × 2 tables and results are given with 95% confidence intervals. Youden’s index was calculated as sensitivity% + specificity% - 100. Receiver operating characteristic curve (ROC) was used to compare the diagnostic performance of different tests and find the best diagnostic threshold for the E6/E7 mRNA expression testing. *p* value < 0.05 was considered as statistical significance for all analysis.

## Results

### Cervical histology results in patients with ASCUS

A total of 300 women with ASCUS underwent cervical histopathology diagnosis. 46.0% (138/300) of ASCUS had cervical inflammation, 36.0% (108/300) had CIN 1, 9.7% (29/300) had CIN2 and 7.3% (22/300) had CIN3. Three cases of cervical squamous cell carcinoma were diagnosed in this study (1.0%). Among them, 54 cases were diagnosed as CIN2+ (18.0%) and 25 cases as CIN3+ (8.3%).

### Test positivity in relation to the histopathologic diagnosis

**Fig. 1:**
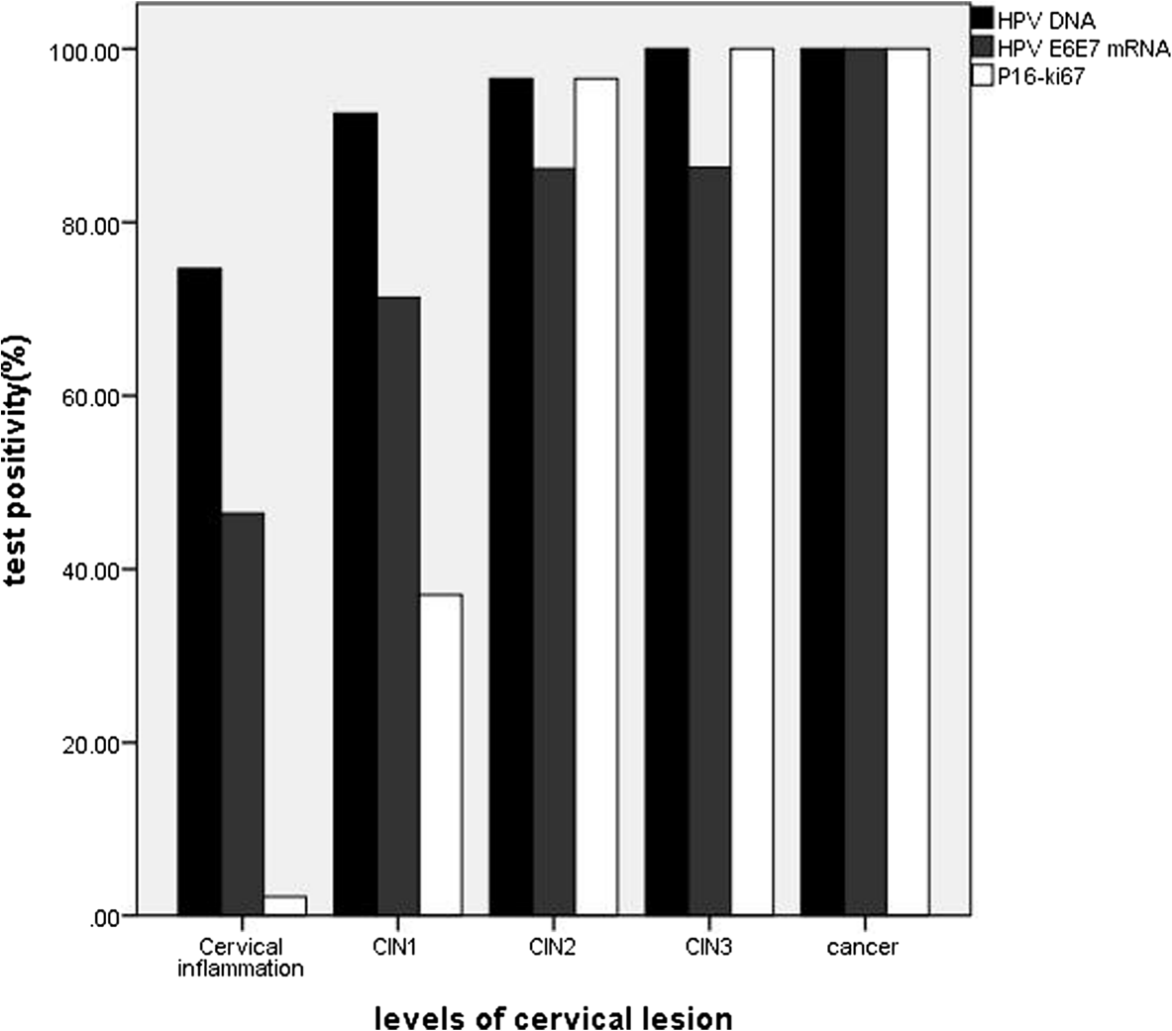
Positive rate of HPV DNA assay, HPV E6/E7 mRNA testing and p16/Ki-67 immunostaining in different levels of cervical lesion. Note: CIN1: grade 1 cervical intraepithelial neoplasia; CIN2: grade 2 cervical intraepithelial neoplasia; CIN3: grade 3 cervical intraepithelial neoplasia

We calculated the positive rate of different methods in different levels of cervical tissue. The overall test positivity was 85.3% (256/300) in HPV DNA assay, 62.7% (188/300) in HPV E6/E7 mRNA testing and 32.0% (96/300) in p16/Ki67 immunocytochemistry. HPV E6/E7 mRNA testing and p16/Ki67 immunocytochemistry were less frequently positive in ASCUS than HPV DNA test. The p16/Ki67 immunocytochemistry positivity was 32.0%, indicating that if we use p16/Ki67 immunocytochemistry instead of HPV DNA assay to triage ASCUS, we will reduce the rate of colposcopy referrals by 53.3% (Table 1). 74.6% (103/138) patients with cervical inflammation were HPV DNA assay positive, 46.4% (64/138) were HPV E6/E7mRNA testing positive and 2.2% (3/138) were p16/Ki67 immunocytochemistry positive. 92.6% (100/108) patients with CIN1 were HPV DNA assay positive, 71.3% (77/108) were HPV E6/E7 mRNA testing positive and 37.0% (40/108) were p16/Ki67 immunocytochemistry positive. 96.6% (28/29) patients with CIN2 were HPV DNA assay positive, 86.2% (25/29) were HPV E6/E7 mRNA testing positive and 96.6% (28/29) were p16/Ki67 immunocytochemistry positive. 100% (22/22) patients with CIN3 were HPV DNA assay positive, 86.4% (19/22) were HPV E6/E7mRNA testing positive and 100% (22/22) were p16/Ki67 immunocytochemistry positive. 100% (3/3) patients with cervical cancer were HPV DNA assay positive, 100% (3/3) were HPV E6/E7 mRNA testing positive and 100% (3/3) were p16/Ki67 immunocytochemistry positive (Fig. 1).

**Table 1:** Positive rate of different methods in different levels of cervical lesion

Test	Referral Rate	Positive rate		*χ*^2^ value *p* value		Positive rate		*χ*^2^ value *P* value	
		CIN1-	CIN2+			CIN2-	CIN3+		
DNA	85.3%	82.5% (203/246)	98.1% (53/54)	8.641	0.003	84.0%(231/275)	100%(25/25)	*	0.033
E6/E7mRNA	62.7%	57.3% (141/246)	87.0% (47/54)	16.717	0.000	60.4%(166/275)	88.0%(22/25)	7.481	0.006
p16/Ki67	32.0%	17.5% (43/246)	98.1% (53/54)	132.421	0.000	25.8%(71/275)	100% (25/25)	57.955	0.000

*The statistical method used is Fisher’s exact probabilities, so χ^2^ value is missing. CIN1-: The diagnoses of cervical inflammation and CIN1 are referred as CIN1-; CIN2+: the diagnoses of CIN2, CIN3 and carcinoma are referred as CIN2+. CIN2-: cervical inflammation, CIN1 and CIN2 are referred as CIN2-. CIN3+: CIN3 and carcinoma are referred as CIN3+. DNA: HPV DNA assay; E6/E7 mRNA: HPV E6/E7 mRNA testing; p16/Ki67: p16/Ki67 immunocytochemistry

HPV DNA positive rate was 82.5% (203/246) in CIN1-, 98.1% (53/54) in CIN2+, and the differences between the lesions were statistically significant (*χ*^2^ = 8.641, *P* = 0.003). This trend was also seen in HPV E6/E7mRNA testing and p16/Ki67 immunocytochemistry, and also seen between CIN2- and CIN3+ (Table 1).

### Comparison of the expression level of HPV E6/E7 mRNA testing in different cervical lesions

**Fig. 2:**
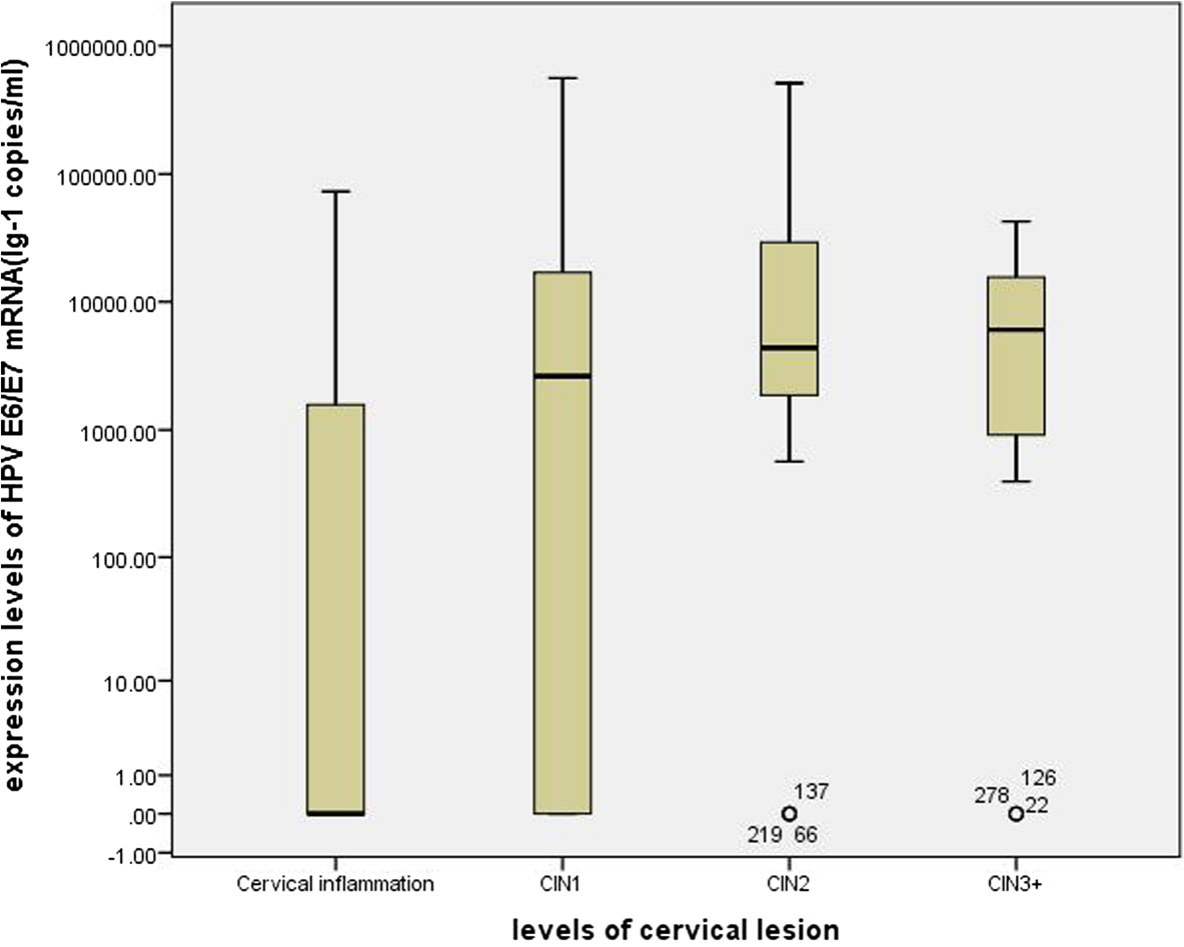
The expression level of HR-HPV E6/E7 mRNA in different levels of cervical lesion. Note: CIN1: grade 1 cervical intraepithelial neoplasia; CIN2: grade 2 cervical intraepithelial neoplasia; CIN3: grade 3 cervical intraepithelial neoplasia; CIN3+: the diagnoses of CIN3 and carcinoma are referred as CIN3+

The expression level of HPV E6/E7 mRNA testing in different cervical lesions is shown in Fig. 2 and Table 2. There were just three cases of cervical cancer, so we combined it with CIN3 to make statistical analysis. The expression levels of E6/E7 mRNA in CIN2+ were higher than in CIN1−, and the differences were statistically significant (*P* < 0.05).

**Table 2:** The results of HPV E6/E7 mRNA expression levels in different levels of cervical lesion

Group	expression levels (copies / ml)^a^	Average rank	*Z* value	*p* value
cervical inflammation	0(0–1586.03)			
CIN1	2643.67(0–17,497.47)			
CIN2	4354.17(1669.85–34,429.90)			
CIN3+	6022.75(866.28–16,847.81)			
CIN1-	560.98(0–4253.46)	139.70	−4.728	< 0.001
CIN2+	4815.42(1011.54–19,392.66)	199.71		

^a^Median of mRNA load, with 25th–75th percentile in parentheses; CIN: cervical intraepithelial neoplasia; CIN1-: The diagnoses of cervical inflammation and CIN1 are referred as CIN1 -; CIN2+: the diagnoses of CIN2, CIN3 and carcinoma are referred as CIN2 +. CIN3+: the diagnoses of CIN3 and carcinoma are referred as CIN3 +

### Diagnostic sensitivity and specificity of the different tests

**Fig. 3:**
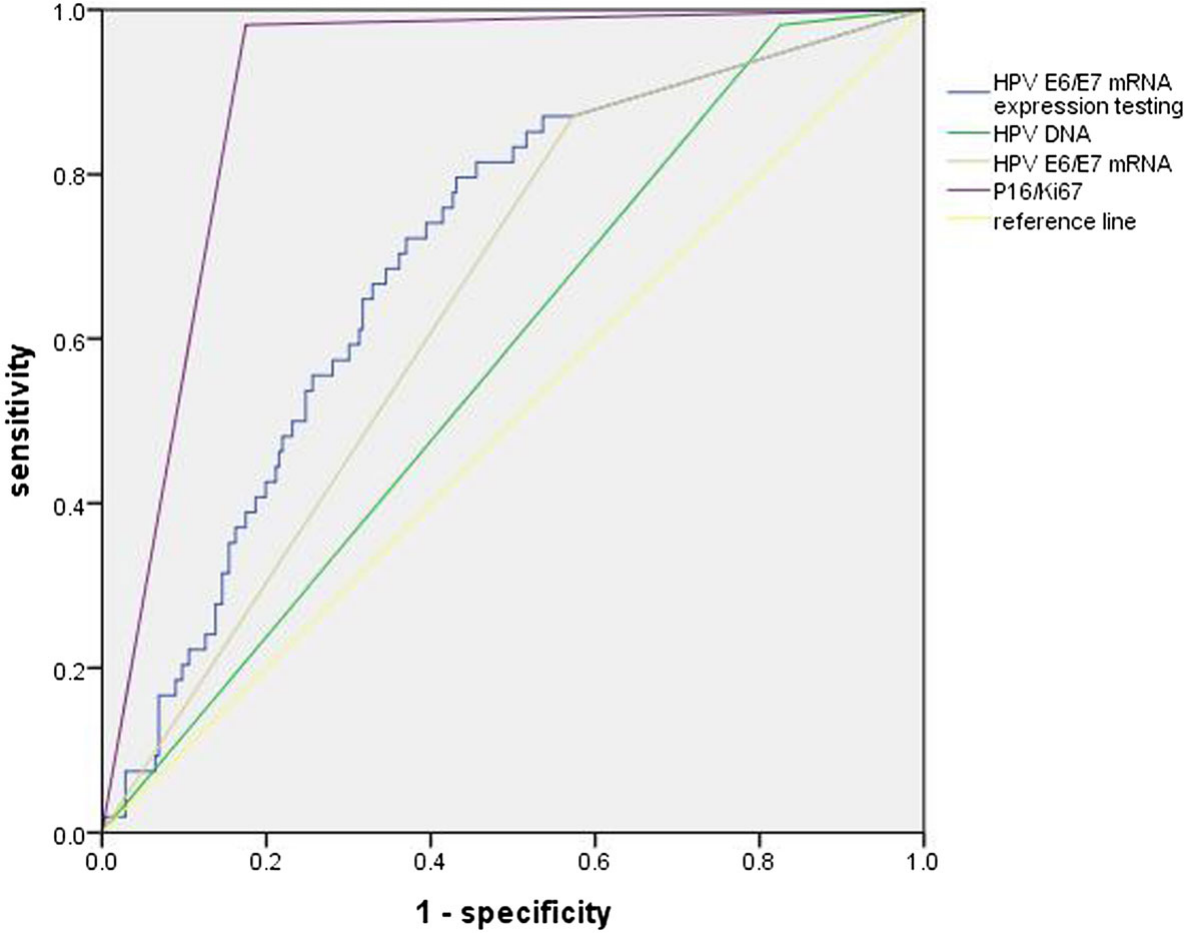
ROC curve of HPV DNA assay, HPV E6/E7 mRNA testing and p16/Ki-67 immunocytochemistry for detecting CIN2+. Note: HPV E6/E7 mRNA test: using 1 copies/ml acting as cut-off value to judge negative and positive of HPV E6/E7 mRNA. HPV E6/E7 mRNA expression: the quantitative detection of HPV E6/E7 mRNA

The sensitivity, specificity, PPV and NPV for CIN2+ and CIN3+ of the HPV DNA assay, HPV E6/E7 mRNA testing and p16/Ki67 immunocytochemistry were shown in Table 3. For endpoint of CIN2+, the sensitivity between the three tests is no statistical difference (*χ*^2^ = 3.375, *P* = 0.066 (HPV DNA assay vs. HPV E6/E7 mRNA testing), *χ*^2^ = 3.375, *P* = 0.066 (HPV E6/E7 mRNA testing vs. p16/Ki67 immunocytochemistry), *χ*^2^ = 0.000 (HPV DNA assay vs. p16/Ki67 immunocytochemistry)), but the specificity is statistical difference (χ^2^ = 37.147, *P* < 0.001 (HPV DNA assay vs. HPV E6/E7 mRNA testing), *χ*^2^ = 83.377, *P* < 0.001 (HPV E6/E7 mRNA testing vs. p16/Ki67 immunocytochemistry), *χ*^2^ = 208.13, *P* < 0.001 (HPV DNA assay vs. p16/Ki67 immunocytochemistry)). ROC curve was used to determine an optimal cut-off value and further demonstrate the diagnostic performance of HPV E6/E7 mRNA testing for detecting CIN2+ (Fig. 3). The expression level of 882.53 copies/ml was the optimal cut-off value for HPV E6/E7 mRNA testing to diagnose CIN2+, and at this time, the sensitivity and specificity was 79.6 and 56.9%. The accuracy of different assay was also displayed in ROC curve by calculating the area under the curve (AUC). Among the three tests, the AUC of p16/Ki67 immunocytochemistry was the largest (Table 4). In this section, we also calculated the sensitivity and specificity of different detection methods in different age groups (Table 5). The sensitivity and specificity of all methods were larger in group with age greater than 30 years old.

**Table 3:** Sensitivity, specificity, PPV and NPV of the different tests

Test	Endpoint	Sensitivity	Specificity	PPV	NPV	Youden
		(95% CI)	(95% CI)	(95% CI)	(95% CI)	
HPV DNA	CIN2+	98.2% (90.2, 99.7)	17.5% (13.2, 22.7)	20.7% (16.2, 26.1)	97.7% (88.2, 99.6)	15.7
	CIN3+	100% (86.7, 100)	16.0% (12.1, 20.8)	9.8% (6.7, 14.0)	100% (92.0, 100)	16.0
HPV E6/E7 mRNA	CIN2+	87.0% (75.6, 93.6)	42.7% (36.7, 48.9)	25.0% (19.4, 31.7)	93.8% (87.7, 96.9)	29.7
	CIN3+	88.0% (70.0, 95.8)	39.6% (34.0, 45.5)	11.7% (7.9, 17.1)	97.3% (92.4, 99.1)	27.6
p16/Ki67	CIN2+	98.2% (90.2, 99.7)	82.5% (77.3, 86.8)	55.2% (45.3, 64.8)	99.5% (97.3, 99.9)	80.7
	CIN3+	100% (86.7, 100)	74.2% (68.7, 79.0)	26.0% (18.3, 35.6)	100% (98.2, 100)	74.2

Note: PPV = positive predictive value; NPV = negative predictive value. Results in % with 95% confidence interval (95% CI)

**Table 4:** The area under ROC curve of different test

Test	AUC	95% CI
HPV DNA	0.578	(0.501–0.655)
HPV E6/E7 mRNA	0.649	(0.575–0.722)
HPV E6/E7 mRNA expression	0.700	(0.628–0.772)
p16/Ki67	0.903	(0.866–0.940)

AUC = the area under the curve. Results in % with 95% confidence interval (95% CI). HPV E6/E7 mRNA expression means that 882.53 copies/ml act as cut-off value to judge negative and positive of HPV E6/E7 mRNA testing and calculate the AUC

**Table 5:** Sensitivity, specificity, PPV and NPV of the different tests in different age group

		Age ≥ 30 years				Age ≥ 30 years		
	N	HPV DNA+	HPV E6/E7 mRNA+	p16/Ki67+	N	HPV DNA+	HPV E6/E7 mRNA+	p16/Ki67+
		n(%)	n(%)	n(%)		n(%)	n(%)	n(%)
Endpoint = CIN2+								
CIN1-	58	53(91.4%)	35(60.3%)	16(27.6%)	188	150(79.8%)	106(56.4%)	27(14.4%)
CIN2+	17	16(94.1%)	13(76.5%)	17(100%)	37	37(100%)	34(91.9%)	36(97.3%)
Sensitivity		94.1%	76.5%	100%		100%	91.9%	97.3%
(95% CI)		(73.0, 99.0)	(52.7, 90.5)	(81.6, 100)		(90.6, 100)	(78.7, 97.2)	(86.2, 99.5)
Specificity		8.6%	39.7%	72.4%		20.2%	43.6%	85.6%
(95% CI)		(3.7, 18.6)	(28.1, 52.5)	(59.8, 82.3)		(15.1, 26.5)	(36.7, 50.8)	(79.9, 89.9)
PPV		23.2%	27.1%	51.5%		19.8%	24.3%	57.1%
(95% CI)		(14.8, 34.4)	(16.6, 41.0)	(35.2, 67.5)		(14.7, 26.1)	(17.9, 32.0)	(44.9, 68.6)
NPV		83.3%	85.2%	100%		100%	96.5%	99.4%
(95% CI)		(43.7, 97.0)	(67.5, 94.1)	(91.6, 100)		(90.8, 100)	(90.1, 98.8)	(96.6, 99.9)
Youden		2.7	16.2	72.4		20.2	35.5	82.9
Endpoint = CIN3+								
CIN2-	68	62(91.2%)	43(63.2%)	26(38.2%)	207	169(81.6%)	123(59.4%)	45(21.3%)
CIN3+	7	7(100%)	5(71.4%)	7(100%)	18	18(100%)	17(94.4%)	18(100%)
Sensitivity		100%	71.4%	100%		100%	94.4%	100%
(95% CI)		(64.6, 100)	(35.9, 91.8)	(64.6, 100)		(82.4, 100)	(74.2, 99.0)	(82.4, 100)
Specificity		8.8%	36.8%	61.8%		18.4%	40.6%	78.3%
(95% CI)		(4.1, 17.9)	(26.3, 48.6)	(49.9, 72.4)		(13.7, 24.2)	(34.1, 47.4)	(72.2, 83.3)
PPV		10.1%	10.4%	21.2%		9.6%	12.1%	28.6%
(95% CI)		(5.0, 19.5)	(4.5, 22.2)	(10.7, 37.8)		(6.2, 14.7)	(7.7, 18.6)	(18.9, 40.7)
NPV		100%	92.6%	100%		100%	98.8%	100%
(95% CI)		(61.0, 100)	(76.6, 97.9)	(91.6, 100)		(90.8, 100)	(93.6, 99.8)	(97.7, 100)
Youden		8.8	8.2	61.8		18.4	35.0	78.3

Note: PPV = positive predictive value; NPV = negative predictive value. Results in % with 95% confidence interval (95% CI). CIN1-: The diagnoses of cervical inflammation and CIN1 are referred as CIN1 -; CIN2+: the diagnoses of CIN2, CIN3 and carcinoma are referred as CIN2 +. CIN2-: cervical inflammation, CIN1 and CIN2 are referred as CIN2-. CIN3+: the diagnoses of CIN3 and carcinoma are referred as CIN3 +

## Discussion

ASCUS is a term that abnormal characteristics of cells are significantly more than inflammatory cell changes, but the number and quality were not enough to diagnose CIN. Frequency of ASCUS lesions is between 1.6 and 9.0% [24]. Our previous research found that Pathology of 160 ASCUS included inflammation, CIN lesions at all levels and cervical cancer [25]. Marwa Fakhreldin et al. [26] retrospectively analyzed 297 cases of ASCUS and found that pathology of ASCUS contained normal cervical tissue, CIN1–3 and cervical cancer. In view of this, if women with ASCUS can’t be treated properly, high grade cervical intraepithelial neoplasia lesions or cervical cancer lesions will be missed. However, as found in this study, CIN2+ just only accounts for 18.0% and CIN3 + for 8.3% and most of ASCUS are CIN1 or cervical inflammation, ASCUS will be overtreated if all women with ASCUS undergo colposcopy. For different tests in our study, the positive rates were statistically different in different cervical lesions, which can be confirmed by many studies [27, 28]. The positivity of HPV DNA test is 85.3%, HPV E6/E7 mRNA testing is 62.7% and p16/Ki67 immunocytochemistry is 32.0%, thus if we use HPV E6/E7 mRNA testing or p16/Ki67 immunocytochemistry instead of HPV DNA assay, the rate of colposcopy referral will be reduced. A low positivity rate can be translated into a low referral rate for colposcopy which is very appealing in a triage setting.

The present study also evaluates the performance of HPV E6/E7 mRNA testing, p16/Ki67 immunocytochemistry and HPV DNA assay in triaging women with ASCUS in a cross-sectional clinical setting. HPV E6/E7 mRNA testing and p16/Ki67 immunocytochemistry were identified in recent studies to increase specificity for the detection of high grade cervical disease compared to HPV DNA detection [29]. In women with HR-HPV-positive ASCUS and LSIL, sensitivity and specificity of p16/Ki67 immunocytochemistry for detection of CIN3 were 90.6 and 48.6%, respectively [22]. Five eligible studies were identified in a meta-analysis, which found that in the ASCUS subgroup, taking CIN2+ as the endpoint, sensitivity ranged from 0.64 to 0.92 (p16/Ki67 test) versus 0.91 to 0.97 (HPV DNA test); specificity ranged from 0.53 to 0.81 versus 0.26 to 0.44, respectively [30]. Nicolas Wentzensen et al. [22] suggested that the sensitivity and specificity of p16/Ki67 immunocytochemistry for detection of CIN2+ were 85.5 and 59.4% respectively and had a sensitivity close to HR-HPV DNA testing *(P* = 0.32), but a higher specificity(*P* = 0.0001). Increasing the p16/Ki67 threshold to two or more dual stain-positive cells led to a substantial reduction in test positivity compared with cytology and the dual stain assay at the usual cutoff. At a cutoff of five or more dual stain-positive cells, the sensitivity for detection of CIN3+ was statistically significantly lower compared with both cytology and the dual stain assay at the usual cutoff, while the specificity and the PPV were statistically significantly increased [31]. In our study, the specificity of p16/Ki67 immunocytochemistry for detection of CIN2+ was higher than HPV DNA test (82.5% vs. 17.5%, χ^2^ = 208.13, *P* < 0.001). These finding support that p16/Ki67 can be a viable option for ASCUS triage. In a review that compared HPV E6/E7 assay versus HPV DNA test (Hybrid Capture 2 method) in triage of women with ASCUS or LSIL cervical cytology, the pooled sensitivity and specificity of HPV E6/E7 assay to triage ASCUS to detect underlying CIN3 or worse was 96.2% (95% CI: 91.7–98.3%) and 54.9% (95% CI: 43.5–65.9%), respectively. HPV E6/E7 assay and HPV DNA test showed similar pooled sensitivity; however, the specificity of the former was significantly higher (ratio: 1.19; 95% CI: 1.08–1.31 for CIN2+) [32]. In our study similarly found that HPV E6/E7 mRNA testing have greater specificity than HPV DNA test (42.7% vs. 17.48%, χ^2^ = 37.147, *P* < 0.001), while the sensitivity is similar (98.2% vs. 87.0%). HPV assays for detecting the mRNA of 5 hrHPV types may reduce the over-diagnosis of women who have minor cytologic abnormalities. However, given the lower sensitivity, women with negative mRNA test results cannot be considered free of CIN2+ and require further surveillance [33]. It is important to consider the trade-off between sensitivity and specificity of the diagnostic test when designing screening algorithms [34]. The data presented in this study confirm these previous observations reported by our previous research that HPV E6/E7 mRNA testing have higher specificity than HPV DNA test (40.3, 95%CI: 32.4–48.8 vs. 15.1, 95%CI: 10.3–22.7) [25]. These results showed greater specificity without loss in sensitivity for HPV E6/E7 mRNA testing in triage of ASCUS compared to HPV DNA assay.

From our study, we can see that both p16/Ki67 immunocytochemistry and HPV E6/E7 mRNA testing have higher specificities than HPV DNA test, when we group analysis by 30 years old, the accuracy of each test is higher in the group over 30 years old. However, we didn’t find a study that solve such a problem: which is better in triaging ASCUS for p16/Ki67 immunocytochemistry and HPV E6/E7 mRNA testing. Our present data suggested that p16/Ki67 immunocytochemistry has a higher sensitivity than HPV E6/E7 mRNA testing (82.5, 95%CI: 77.3–86.8 vs. 42.7, 95%CI: 36.7–48.9). ROC curve was used to further demonstrate the diagnostic performance of p16/Ki67 immunocytochemistry, HPV E6/E7 mRNA testing and HPV DNA test for detecting CIN2+, we can see that the AUC of p16/Ki67 immunocytochemistry is the largest (AUC = 0.903, 95%CI: 0.866–0.940). The figure found that when the optimal cut-off value of HPV E6/E7 mRNA testing is selected at 882.53 copies/ml, the specificity of HPV E6/E7 mRNA testing is improved. Tong-Yu Liu’s and Ye-li Yao’s finding also supported this result [23, 35]. However, the diagnostic performance of HPV E6/E7 mRNA testing was still lower than p16/Ki67 immunocytochemistry. Miriam Reuschenbach et al. [28] conducted a clinical experiment which compare the accuracy of CINtec p16INK4a-cytology, HPV E6/E7 mRNA and HPV DNA, they found that the specificity to detect high grade dysplasia was highest for CINtec p16INK4a-cytology (60.6% (52.7–68.0) in CIN3+ and 74.8% (65.5–82.3) in CIN2+), followed by HPV E6/E7 mRNA (56.4% (48.4–64.0) in CIN3+ and 71.2% (61.7–79.2) in CIN2+) and HPV DNA (49.1% (41.3–56.9) in CIN3+ and 63.4% (53.7–72.1) in CIN2+). As we all know, some normal cells may express p16, observation of p16-positive cells in cytology preparations requires additional morphologic evaluation to achieve adequate specificity. An assay of p16/Ki67 has been developed that combines staining for p16 with staining for the proliferation marker Ki67 on cytological slides. Theoretically, co-expression of p16 and Ki67 in the same cell should indicate HPV-related transformation obviating the need for morphological interpretation and the diagnostic accuracy of p16/Ki67 is higher than p16 alone. In summary, we can give the answer that p16/Ki67 immunocytochemistry may be better in triaging ASCUS than HPV E6/E7 mRNA testing.

## Conclusions

Both biomarker tests, HPV E6/E7 mRNA testing and p16/Ki67 immunocytochemistry, especially p16/Ki67 immunocytochemistry, could be a valuable test in such a setting with higher specificity for the detection of high-grade cervical neoplasia than HPV DNA detection without losing sensitivity. p16/Ki67 immunocytochemistry is promising to be used for the efficient detection of cervical precancer and cancers in triaging women with ASCUS. However, although our results are gratifying, further cost-effectiveness analyses have to be considered when implementing the two strategies for cervical cancer prevention.

## Abbreviations

ASCUS: Atypical squamous cell of undetermined significance
AUC: Area under the curve
CIN1: Mild cervical intraepithelial neoplasia
CIN2: Moderate cervical intraepithelial neoplasia
CIN3: Severe cervical intraepithelial neoplasia
HR-HPV: High-risk human papillomaviruses
Pap: Papanicolaou
ROC: Receiver operating characteristic
